# Myalgic Encephalomyelitis/Chronic Fatigue Syndrome is common in post-acute sequelae of SARS-CoV-2 infection (PASC): Results from a post-COVID-19 multidisciplinary clinic

**DOI:** 10.3389/fneur.2023.1090747

**Published:** 2023-02-24

**Authors:** Hector Bonilla, Tom C. Quach, Anushri Tiwari, Andres E. Bonilla, Mitchell Miglis, Phillip C. Yang, Lauren E. Eggert, Husham Sharifi, Audra Horomanski, Aruna Subramanian, Liza Smirnoff, Norah Simpson, Houssan Halawi, Oliver Sum-ping, Agnieszka Kalinowski, Zara M. Patel, Robert William Shafer, Linda N. Geng

**Affiliations:** ^1^Department of Medicine, Stanford University, Stanford, CA, United States; ^2^Department of Molecular, Cell and Developmental Biology, University of Michigan, Ann Arbor, MI, United States

**Keywords:** PASC, ME/CFS, symptoms, post-COVID-19 clinic, prevalence

## Abstract

**Background:**

The global prevalence of PASC is estimated to be present in 0·43 and based on the WHO estimation of 470 million worldwide COVID-19 infections, corresponds to around 200 million people experiencing long COVID symptoms. Despite this, its clinical features are not well-defined.

**Methods:**

We collected retrospective data from 140 patients with PASC in a post-COVID-19 clinic on demographics, risk factors, illness severity (graded as one-mild to five-severe), functional status, and 29 symptoms and principal component symptoms cluster analysis. The Institute of Medicine (IOM) 2015 criteria were used to determine the Encephalomyelitis/Chronic Fatigue Syndrome (ME/CFS) phenotype.

**Findings:**

The median age was 47 years, 59.0% were female; 49.3% White, 17.2% Hispanic, 14.9% Asian, and 6.7% Black. Only 12.7% required hospitalization. Seventy-two (53.5%) patients had no known comorbid conditions. Forty-five (33.9%) were significantly debilitated. The median duration of symptoms was 285.5 days, and the number of symptoms was 12. The most common symptoms were fatigue (86.5%), post-exertional malaise (82.8%), brain fog (81.2%), unrefreshing sleep (76.7%), and lethargy (74.6%). Forty-three percent fit the criteria for ME/CFS, majority were female, and obesity (BMI > 30 Kg/m^2^) (*P* = 0.00377895) and worse functional status (*P* = 0.0110474) were significantly associated with ME/CFS.

**Interpretations:**

Most PASC patients evaluated at our clinic had no comorbid condition and were not hospitalized for acute COVID-19. One-third of patients experienced a severe decline in their functional status. About 43% had the ME/CFS subtype.

## Introduction

While most patients recover within weeks of SARS-CoV-2 infection, others experience debilitating symptoms that persist beyond the acute period (1). The overall global prevalence of post-COVID-19 conditions is estimated at 0.43 of acute cases (hospitalized 0.54 and non-hospitalized 0.36) (2). These post-COVID conditions, collectively known as a Post-Acute Sequelae of SARS-CoV-2 infection (PASC), or long COVID, are increasingly recognized even in patients who experience asymptomatic or mild SARS-CoV-2 infection (3). The Center for Disease Control and Prevention (CDC) defines post-COVID conditions as symptoms persisting beyond 28 days after infection (4), while the UK National Institute for Health and Care Excellence (NICE) (5) and the World Health Organization (WHO) define this syndrome as symptoms persisting beyond 12 weeks after infection (6). The American Academic of Physical Medicine and Rehabilitation (AAPMR&R) estimates that there are more than 24 million cases of long COVID as of May, 2022 (7).

PASC is most often characterized by extreme fatigue exacerbated by exertion, referred to as post-exertional malaise, difficulty with concentration and memory often referred to as brain fog, sleep disturbances, headaches, chest pain, and shortness of breath (2, 8). PASC can range from mild to severe and incapacitating, interfering with patients' daily activities and work requirements (2, 8).

In an online, multi-national survey of 3,762 participants with confirmed or suspected COVID-19, fatigue, post-exertional malaise, and brain fog were the most frequently reported symptoms six months after SARS-CoV-2 infection (8). This cluster of symptoms shares similar features with Encephalomyelitis/Chronic Fatigue Syndrome (ME/CFS), an often-debilitating disease that has a worldwide prevalence close to one percent, is also believed to frequently arise following a viral infection (9, 10). Women are affected 1.5 to 2 times more often than men (2). In two small cohorts of 41 and 42 COVID-19 patients ~45% met the ME/CFS diagnostic criteria (11, 12). The pathobiological mechanism of ME/CFS is not known, but the large number of PASC patients presenting with features of this syndrome may allow us to better understand both conditions. The goals of our study are 2-fold: 1. Characterize our clinical cohort in terms of demographics and clinical presentations. 2. Estimate the prevalence of ME/CFS phenotype in PASC based on the IOM ME/CFS criteria (9).

## Methods

### Study design and participants

The Stanford referral Post-Acute COVID-19 Syndrome (PACS) Clinic is a multidisciplinary center designed to provide clinical expertise in post-COVID-19 conditions, standardization of data collection and clinical management, and integration of research efforts. We standardized the clinical assessment by developing a clinic template embedded in the Electronic Health Records (EHR), allowing the extraction of the same data retrospectively. We used Research Electronic Data Capture (REDCap) and Microsoft Excel platforms for data collection and analysis. The study was approved by the Stanford University Institutional Review Board.

One hundred-forty consecutive adult patients with a history of COVID-19 were seen in the Stanford PACS clinic between May 18, 2021, and February 1, 2022. Referral criteria included a history of symptomatic SARS-CoV-2 infection, a diagnostic test for SARS-CoV-2 by either PCR, antigen detection, or a positive serology before SARS-CoV-2 vaccination, and persistent symptoms for at least 28 days following infection (CDC definition). In addition, each patient's initial symptoms during the acute infection was obtained from the questionnaire before their scheduled clinic visit and from the patient's EHR. The questionnaire includes: (i) the 29 symptoms reported to occur commonly in patients with acute COVID-19 (Supplementary material) to capture the different COVID-19 conditions such as ME/CFS, autonomic, respiratory, cardiac, neurological, psychiatric, olfactory, and gastrointestinal disorders; (ii) the severity of each symptom based on the Likert scale (1 mild symptom and 5 severe) (iii) SARS-CoV-2 vaccination status; (iv) the modified Post COVID-19 Functional Status Scale (FSS) which classifies patients as either asymptomatic (level 1), symptomatic without limitations (level 2), symptomatic with reduced daily activity (level 3), symptomatic with a struggle to perform daily activities (level 4), or incapacitated and bedridden (level 5) (2, 13). From the EHR, we extracted the following: (1) demographics including age, sex, and self-identified race/ethnicity; (2) laboratory results and radiological data obtained before the initial office visit; and (3) vital signs, body mass index (BMI), oxygen saturation, orthostatic blood pressure, heart rate measurements obtained at the initial visit.

**Figure 1:**
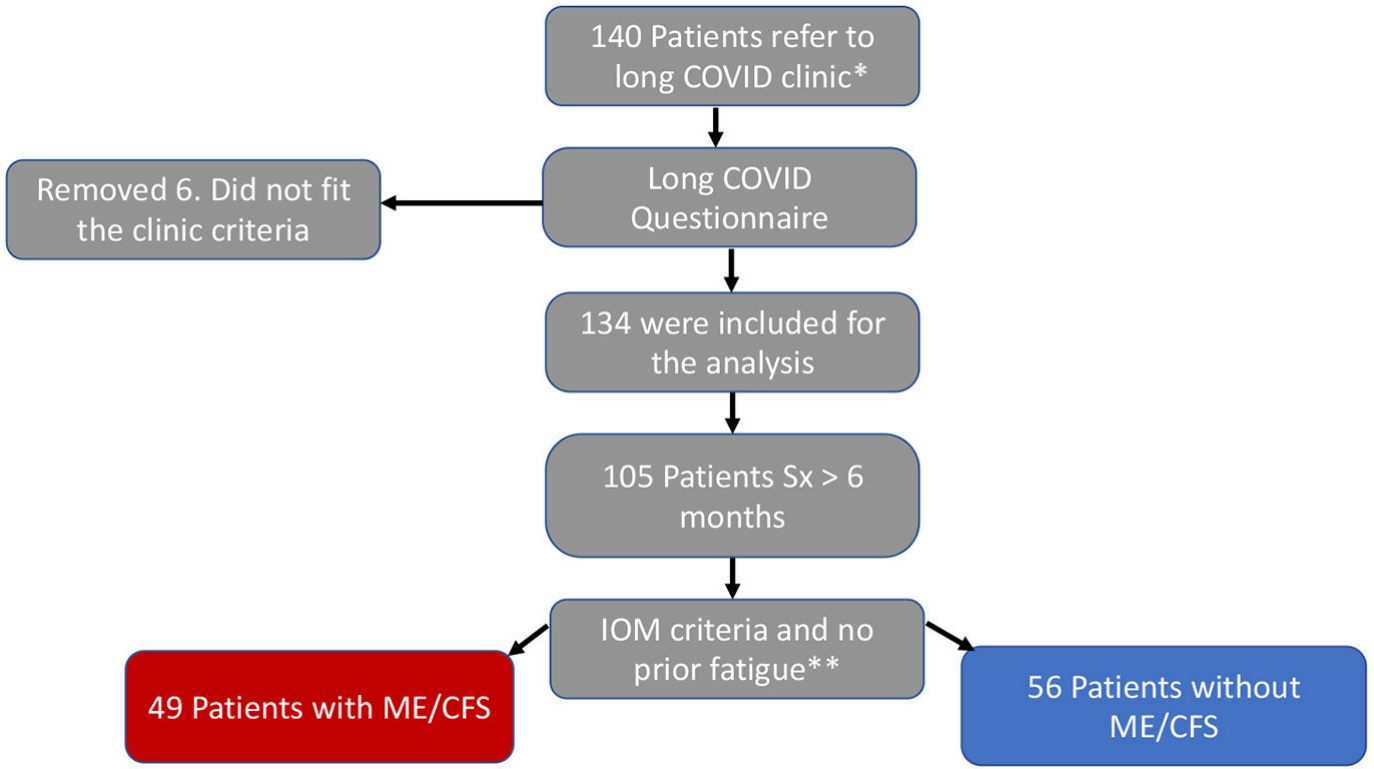
Flow chart. *Positive SARC-CoV-2 test and over 28 days with symptoms. **Severe fatigue, unrefreshing sleep, PEM, and brain fog or orthostatic intolerance.

A second and identical questionnaire was sent to each patient before the clinic visit, collecting the same information in the past seven days as we did for the acute infection (Supplementary material). The Institute of Medicine (IOM) 2015 diagnostic criteria was used to identify the ME/CFS cohort; the patients must have persistent symptoms for at least 6 months; severe and incapacitating fatigue (Likert severity scale 4, or 5), unrefreshing sleep, post-exertional malaise (PEM), and orthostatic intolerance (lightheadedness), or brain fog (9). The ME/CFS cohort were patients who fit the IOM diagnostic criteria, and their clinic records were review to exclude those with a history of fatigue symptom before COVID-19 (Figure 1, flow chart).

### Statistical analysis

To explore how the symptoms correlated with each other in our study cohort, we utilized R (version 3.6.1) cluster analysis algorithms in 134 post-COVID-19 patients based on principal components analysis (PCA) to visualize the distribution of symptoms (14, 15). We used the Wilcoxon Rank Sum Test to calculate the difference in the number of symptoms between males and females, Chi-square to calculate the difference in two categorical variables symptoms, *T*-test to compare the differences between groups and the Spearman Coefficient Correlation (rho) to compare the frequency of the symptoms and the median severity.

## Results

Among the 140 patients referred to the Stanford PASC clinic, six patients were excluded because of lack of a diagnostic test for SARS-CoV-2 infection or an incomplete questionnaire. For the remaining 134 patients, the median age was 47 years old (IQR: 30–60; range: 20–88). Seventy-nine (59%) were female, 66 self-identified as White (49.3%), 23 as Hispanic (17.2%), 20 as Asian (14.9%), and 9 as Black (6.7%). Seventeen (12.7%) required hospitalization for their acute infection and were considered to have had severe disease. Two of these patients required Intensive Care Unit (ICU) admission. Sixty-two (46.3%) patients had a comorbid condition known to predispose to severe disease including obesity (BMI > 30 kg/m^2^) 47 (35.1%), hypertension 24 (17.9%), chronic lung disease 16 (11.9%), diabetes 9 (6.7%), immunosuppressive therapy 5 (3.7%) and/or cardiovascular disease 4 (3%). One hundred and nine (82%) patients had some degree of functional limitation (i.e., FSS of 3, 4, or 5), including 45 (33.9%) who exhibited significantly compromised wellbeing (FSS of 4 or 5) (Table 1).

**Table 1:** Patient's characteristics and distribution PASC cohort and chronic fatigue cohort.

	**PASC cohort (*N* = 134)**	**Chronic fatigue cohort (*****N*** = **105) persistent symptoms over 6 months**		
**Characteristics**		**ME/CFS**	**No ME/CFS**	**Total—** ***P*** **-value**
Age (years) median (range)	47 (20–88)	50 (23–75)	47 (21–88)	47 (21–88), 0.26755835
Sex: female/male (female %)	79/55 (59%)	26/19 (58%)	42/18 (70%)	68/38, 0.48175231
Days post-COVID median (range)	285.5 (34–792)	318 (183–686)	310 (149–792)	310 (183–792), 0.13233956
**Diagnostic test** ***N*** **(%)**				
Molecular/PCR	119 (88.8%)	38 (84%)	54 (90%)	92
Antigen	5 (3.7%)	2 (6%)	3 (5%)	5
Antibodies (+) before Vaccination	10 (7.5%)	5 (10%)	3 (5%)	8
**Treatment**				
Ambulatory	117/134 (87.3%)	44 (98%)	57 (95%)	101 (96%)
Hospital	17/134 (12.7%)	1 (2%)	3 (5%)	4 (4%)
**Functional status (%)**				0.0110474^*^
V (%)	9 (6.8%)	5 (11%)	3 (5%)	8
IV (%)	36 (27.1%)	19 (42%)	13 (22%)	32
III (%)	64 (48.1%)	20 (44%)	27 (45%)	47
1–II (%)	20 (15%)	1 (3%)	17 (28%)	18
**Co-morbid condition** ***N*** **(%)**				0.34714359
None	72 (53.7%)	17 (38%)	36 (60%)	53
One	37 (27.6%)	13 (29%)	18 (30%)	31
Two	15 (11.2%)	7 (15%)	5 (8%)	12
Three	7 (5.2%)	9 (18%)	1 (2%)	10
**BMI kg/m**^2^ **(median, range)**				0.00377895^*^
All	26.2 (16.9–46.6)	29.23 (18.9–47.9)	25.6 (18.3–41.1)	27.2 (16.9–46.6)
>30 (%)	31 (33.6%)	22 (49%)	17 (28%)	39
>35 (%)	21 (15%)	10 (22%)	8 (13%)	19
**Race/ethnicity**				
White	66 (49.3%)	17 (38%)	34 (57%)	51
Hispanic	23 (17.2%)	13 (28%)	7 (12%)	20
Black	9 (6.7%)	2 (4%)	4 (7%)	6
Asian	20 (14.9%)	4 (8%)	8 (13%)	12
Others	2 (1.4%)	1 (2%)	1 (1%)	2
No data	15 (11%)	8 (18%)	6 (10%)	14
**Total**	134 (100%)	45 (43%)	60 (57%)	105 (100%)

PASC, Post-Acute Sequela of SARS-CoV-2 infection; ME/CFS, Myalgic Encephalomyelitis/Chronic Fatigue Syndrome.

* Statistical significant.

The duration of the symptoms at the time of the initial office visit ranged from 34 to 792 days with a median duration of 285.5 days. Of the 29 symptoms assessed in the study, the median number per patient was 12 (range: 1–25 symptoms). The most common symptoms were fatigue (86.5%), post-exertional malaise (82.8%), brain fog (81.2%), unrefreshing sleep (76.7%), and daytime sleepiness (74.6%). The median number of symptoms in female patients was greater than the median number in male patients (13 vs. 10, *p* = 0.005). The most common symptoms significantly higher in the female population were fatigue, insomnia, and change in taste or dysgeusia (*P*-values = 0.001, 0.044, and 0.01, respectively) (Table 2). There was a significant correlation between the frequency and the median severity of the symptom (Correlation coefficient is 0.86, *P* < 0.001).

**Table 2:** Distribution rates of the most common 15 symptoms in PASC patients by sex and severity.

**Symptom**	**Rates by sex (%)**		***P*** **-value**	Rates Likert scale 4–5^**^	Total (%)^**^
	**Female**	**Male**			
Fatigue	88.6	81.8	0.0011^*^	61.8	86.5
Post exertional malaise	84.8	80	0.4675	66.6	82.8
Brain fog	81	80	0.8840	50	81.2
Unrefreshing sleep	81	69.1	0.1113	49	76.7
Lethargic	78.5	69.1	0.2191	57	74.6
Insomnia	74.7	58.2	0.0441^*^	44	65.9
Headache	65.8	63.6	0.9129	31.4	62.1
Anxiety/depression	57	67.3	0.3226	39.5	52.6
Lightheadedness	57	45.5	0.1895	32.8	50.8
Gastrointestinal	57	40	0.1289	20.9	48.9
Shortness of breath	51.9	41.8	0.3145	30.1	43.2
Nasal congestion	49.4	32.7	0.07590	21.4	42
Changes of smell	49.4	32.7	0.1024	32.7	41.2
Changes of taste	46.8	27.3	0.01^*^	31.5	39.2
Cough	44.3	29.1	0.074	23.5	36.6

* *P*-value < 0.05 Chi-square.

** Pearson correlation coefficient 0.92, *P*-value < 0.0001.

In the PCA analysis, the direction of arrows in the correlation circle illustrates the extent to which symptoms were likely to occur in the same patient while the factor map indicates the contribution of different symptoms to the first five components (**Figure 3**). The analysis suggests that fatigue, PEM, daytime sleepiness (lethargy), brain fog, and unrefreshing sleep were likely to occur together. These symptoms were major contributors to the first component while anosmia and nasal congestion were major contributors to the second component. Similar analysis was performed on 72 patients without pre-COVID comorbidities two main groups were identified one with anosmia, headache, and congestion and a second that includes unrefreshing sleep, fatigue, and lethargy.

**Figure 2:**
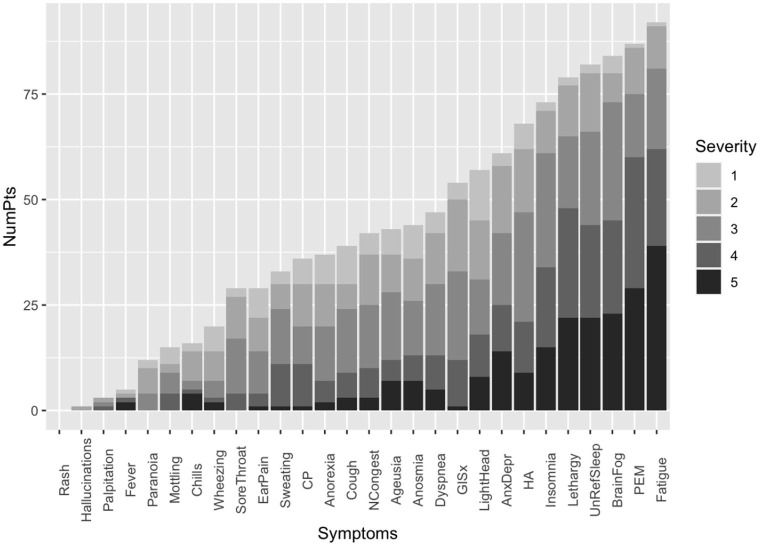
Distribution of the frequency and severity of the symptoms on the PASC clinic intake questionnaire for subgroup of 105 patients with persistent symptoms for six or more months. Symptom severity was measured on the Likert scale with a severity score of 5 being the most severe. CP, Chest pain; GISx, Gastrointestinal symptoms; Anex/Depr, Anxiety/Depression; HA, Headache; UnRefreSleep, Unrefreshed sleep; PEM, Post-Exertional Malaise; NCongest, Nasal congestion.

**Figure 3:**
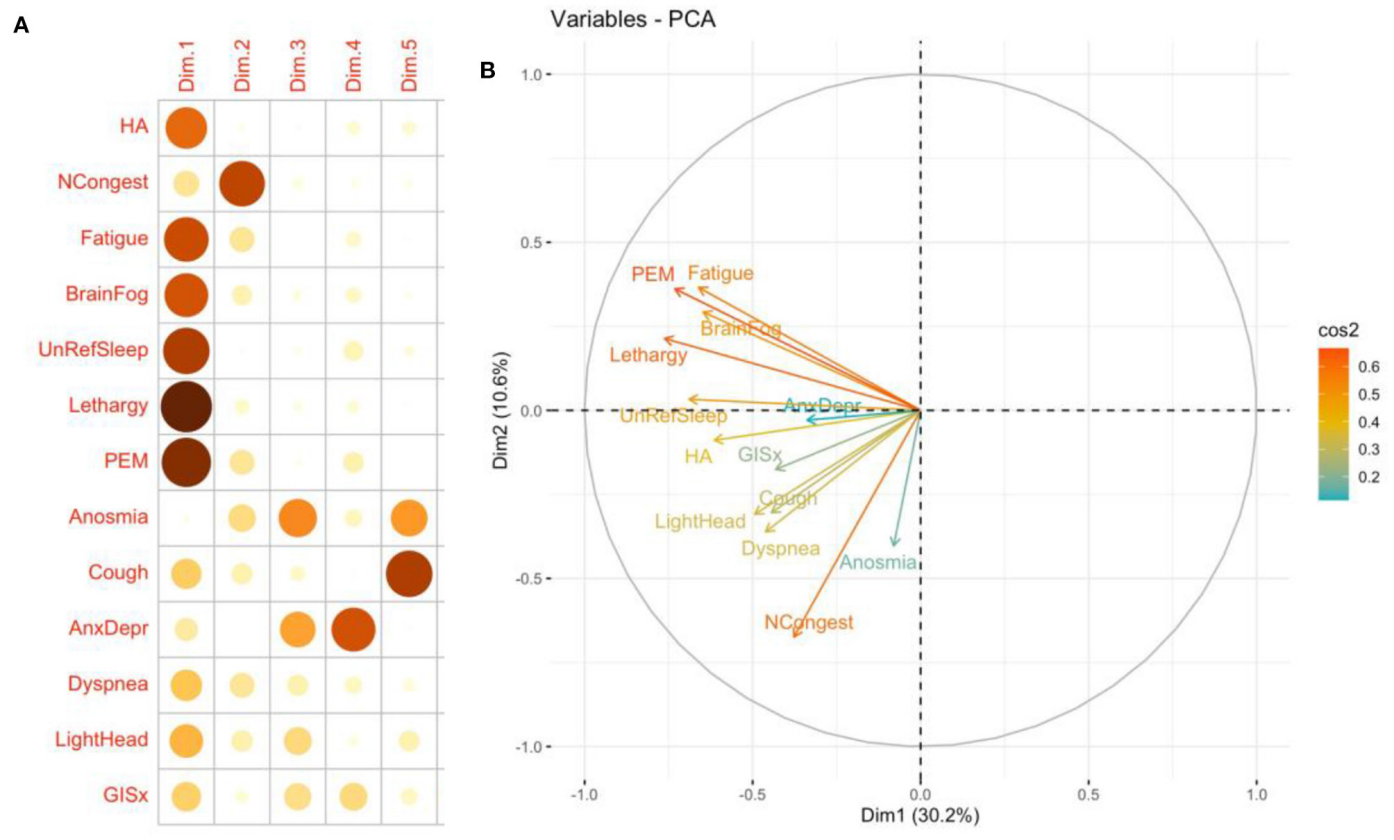
Quality of representation of 13 most common symptoms mapped to the first five dimensions **(A)** and principal components analysis correlation circle **(B)**. Insomnia and ageusia were not included in the analysis. The closer the variable to the correlation circle, the better the representation on the factor map. The quality of the representation of the symptom on the first five dimensions is measured by the squared cosine between the symptom vector and its projection on the dimension. The proportions on the left side of the factor map represent a color scale (14). CP, Chest pain; GISx, Gastrointestinal symptoms; Anex/Depr, Anxiety/Depression; HA, Headache; Lethargy, daytime sleepiness; UnRefrSleep, Unrefreshed sleep; PEM, Post-Exertional Malaise.

We assessed the prevalence of the ME/CFS phenotype in 105 patients (69%) who had persistent symptoms longer than 6 months. The most common symptoms were clustered in the ME/CFS (Fatigue, PEM, brain fog, unrefreshing sleep, and lethargy) and autonomic dysfunction categories (lightheadedness and gastrointestinal) (**Figure 3**). The top six most common and severe symptoms were fatigue, PEM, brain fog, unrefreshing sleep, daytime sleepiness, and insomnia (Figure 2). Forty-five (43%) of the study cohort fulfill the IOM criteria for ME/CFS (Table 1). Like our PASC cohort, the ME/CFS cohort was predominantly female, non-hospitalized, and healthy individuals and obesity was the most common risk factor (BMI > 30 Kg/m^2^) (Table 1). But obesity and worse FSS (FFS 4 and 5) were significantly higher in the ME/CFS population (*P* = 0.00377895; *P* = 0.0110474, respectively) (Table 1).

## Discussion

This manuscript presents the characteristics of 134 patients referred to a long COVID clinic who had who have history of SARS-CoV-2 infection and more than 28 more days of symptoms. The median age in our cohort was 47 years and ranged from 20 to 88 years, with a female predominance of 59%. Similar age and sex distribution was reported in several other studies (2, 16). In contrast to hospitalized patients, females PASC were represented in a lower proportion, less severe symptoms, and lower mortality than males (17–19). These differences in sex distribution could be explained by the variations in the immune response between males and females, and the fact that female patients have more robust inflammatory, antiviral, and humoral immune responses, biological differences on sex hormones, and expression and regulation of angiotensin-converting enzyme 2 (ACE2) (18, 20). Our study cohort, similar to other PASC studies, the subjects were predominantly white females, with obesity (17, 21, 22) characteristics associated with lower likelihood for full recovery (23). In contrast, some racial and ethnic minority groups, such as Native American Indians, Alaska Natives, Hispanic and Black, have been shown to have a disproportionately higher risk for infection, severity of illness, hospitalization, and deaths. Those groups were underrepresented in our study (24).

The large majority (87%) of our PASC patients had not been hospitalized or required oxygen for COVID-19 disease. The prevalence of PASC in asymptomatic patients with the mild disease is reported between 30 and 60% (3, 25). We can postulate that unknown factors besides hypoxemia and hospitalization are the drivers of PASC symptoms such as virus persistence, overactivation of the immune system, amyloid fibrin microclots, auto-antibodies, virus reactivation and others, may play a role in the pathobiology of this illness (26).

In our study, the duration of symptoms ranged from 34 to 792 days, with a median duration of 285.5 days. Females experienced more symptoms than males. The most common symptoms were fatigue, post-exertional malaise, brain fog, unrefreshing sleep, and daytime sleepiness; and the frequency of the symptoms correlated with severity (Figure 2, Table 2) and the ME/CFS symptoms clustered with one another (Figure 3). A reliable and straightforward long COVID score system is needed in post-COVID research and clinical care. In a population-based cohort study of confirmed SARS-CoV-2 infection, higher severity scores correlate with lower health quality of life (27). In a meta-analysis which found that fatigue/weakness, myalgia/arthralgia, depression, anxiety, memory loss, concentration difficulties, dyspnea, and insomnia, were the most prevalent symptoms (2, 28). Fatigue was the most prevalent symptom across the PASC studies (2, 28). Post-viral fatigue was commonly reported after a viral infection such as Influenza, Severe acute respiratory syndrome/Middle East respiratory syndrome coronavirus (SARS/MERS), and Ebola (29).

When we compare the frequency of the symptoms and severity, we found a significant correlation between the frequency and the severity of the symptom (Likert scale 4 and 5). A reliable and simple long COVID score system is tool need in post COVID research and clinical care. It will be important to access clinical outcomes and evaluated therapeutics interventions. In a population-based cohort study confirmed SARS-CoV-2 infection, Bahmer et al., the higher score in severity of COVID symptoms correlated with lower health quality of life (27).

PCA is often used to transform a large set of variables into a smaller one that contains most of the information in the large set. Smaller data sets are easier to explore and visualize and facilitate analyzing data much easier and faster (15). We used it to cluster symptoms and severity. We were able to visualize a group that resembles ME/CFS.

In a study on 233 SARS survivors, 40.3 % reported having chronic fatigue, and 27.1% met the criteria for ME/CFS; after influenza with H1N1, ME/CFS has reported 2.08 cases/100,000 person-month (29). In our study, 43% of the selected cohort fulfilled all the ME/CFS criteria, which is similar to 45% reported by Mancini et al. (11) and Kedor et al. (12). Like ME/CFS, the ME/CFS-PASC phenotype was more prevalent among the non-hospitalized female population (Table 1). The clinical similarities in our study cohort between ME/CFS and ME/CFS-PASC allow us to suggest common pathobiology. Those similarities include a preceding a viral illness (30), increase in inflammatory cytokines, neuroinflammatory change, mitochondria dysfunction, and alteration in NK cell function (31). Obesity (BMI > 30 Kg/m^2^) was in our PASC study cohort, and the PASC-ME/CFS is the most common risk factor for this illness that has also been recognized in multiple studies as a risk factor for Long COVID (32, 33). In addition, PASC-ME/CFS was an indicator worse functional status. However, we were not able to confirm that all symptoms resulted from prior SARS-CoV-2 infection and that it is possible that many of the reported symptoms, including chronic fatigue had other contributing causes. We believe there is an important relationship between Obstructive Sleep Apnea (OSA) and COVID-19, with significant symptomatic overlap between these two conditions and emerging data suggesting COVID is a risk factor for OSA, increasing the risk for severity and hospitalization (34, 35). For these reasons, we agree that the relationship between long COVID and OSA deserves to be further explored, though this exploration is beyond the intended scope of this study.

Our study has several limitations. First, this study only represents the experience of a single center in Northern California located in a generally affluent area with a bias toward specific populations. The selection of this cohort may skew our population in the follow ways: (1) this is a referred population with multiple and more severe symptoms (2) our clinics have a lower proportion of underrepresented minority populations. Therefore, a multicenter study that includes a more diverse and larger population is necessary to corroborate our findings.

In summary, about half of the PASC patients with more than 6 months of symptoms fulfilled the ME/CFS criteria, and PASC-ME/CFS is an indicator of worse functional status; the majority of patients seen in our PACS clinic were female, with a median age of 47 years old, with obesity as the most common comorbidity. The majority of the patients had mild to moderate acute infection and were healthy prior to their COVID infection. Fatigue, post-exertional malaise, brain fog, unrefreshing sleep, and daytime sleepiness were the most prevalent and severe symptoms. This commonality between ME/CFS and ME/CFS-PASC may suggest a shared pathobiology. Therefore, defining specific subtypes within the umbrella of PASC/post-COVID conditions can help us understand different pathogenic mechanisms to tailor treatment.

## Data availability statement

The original contributions presented in the study are included in the article/Supplementary material, further inquiries can be directed to the corresponding author.

## Ethics statement

The studies involving human participants were reviewed and approved by Stanford University IRB. Written informed consent for participation was not required for this study in accordance with the national legislation and the institutional requirements.

## Author contributions

HB: conception and design of the study, data collection, analysis, draft, editing of the manuscript, full access to the whole data, and final version approval. TQ, AT, and AB: data collection, full access to the whole data, analysis, draft, and editing of the manuscript. MM, PY, LE, AS, LS, NS, HH, ZP, HS, AH, OS-p, and AK: conception and design of the study, full access to the whole data, and editing of the manuscript. RS and LG: conception and design of the study, full access to the whole data, data collection, analysis, draft, editing of the manuscript, and final version approval. All authors contributed to the article and approved the submitted version.
