# Supplementary material for: Myalgic Encephalomyelitis/Chronic Fatigue Syndrome is common in post-acute sequelae of SARS-CoV-2 infection (PASC): Results from a post-COVID-19 multidisciplinary clinic
Source: Front Neurol. 2023 Feb 24;14:1090747. doi: 10.3389/fneur.2023.1090747 (PMC9998690; doi:10.3389/fneur.2023.1090747)
Supplement: Supplementary file 1 [file Data_Sheet_1.PDF]

First assessment Post Covid infection

Name: ____________________________________ Date of Birth: __________

COVID-19 Symptoms: Start date _____________ Duration of Symptoms (days)___________

Date of Positive Test *(if not done at Stanford, please provide copy of the results)*: _____________

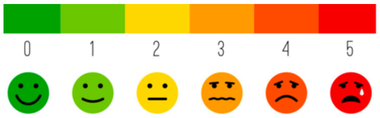

What were your symptoms during the initial COVID-19 infection? Please also select severity.

**Symptoms during acute COVID-19 Infection**	Yes	No	**Severity (1=mild, 5=severe)**
Fever			
Chills			
Headache			
Decrease appetite			
Nose congestion			
Sore throat			
Fatigue			
Brain fog or confusion			
Unrefreshing sleep			
Difficulty sleeping			
Daytime sleepiness			
More fatigue with activity			
Change in smell			
Change in taste			
Ear pain			
New anxiety or depression			
Paranoid thoughts			
Hallucinations			
Cough			
Chest pain			
Difficulty breathing at rest			

Difficulty breathing while walking			
Wheezing			

Lightheadedness on standing			
Fainting spells			
Changes in sweating (more or less)			
Nausea, vomiting, or diarrhea, bloating or constipation			
Changes in color of hands or feet			
Urinary difficulties			

*modified scale from reference: Klok FA, Boon GJAM, Barco S, et al. The Post-COVID-19 Functional Status scale: a tool to measure functional status over time after COVID-19. Eur Respir J 2020; 56: 2001494

New Patient Questionnaire – Post-COVID Infection

(Please complete within 7 days of the office visit)

Name: _________________________________ Date of Birth: _______________

COVID-19 Symptoms: Start date ____________ Duration of Symptoms (days): __________

Date of Positive Test *(if not done at Stanford, please provide copy of the results)*: ________________

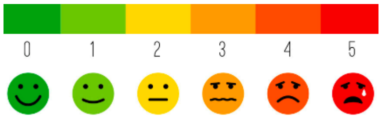

What symptoms are you currently experiencing? Select severity from scale of 1 to 5.

**Current symptoms**	Yes	No	Severity (1=mild, 5=severe)
Fever			
Chills			
Headache			
Decrease appetite			
Nose congestion			
Sore throat			
Fatigue			

Brain fog or confusion			
Unrefreshing sleep			
Difficulty sleeping			
Daytime sleepiness			
More fatigue with activity			
Change in smell			
Change in taste			
Ear pain			
New anxiety or depression			
Paranoid thoughts			
Hallucinations			

Cough			
Chest pain			
Difficulty breathing at rest			
Difficulty breathing while walking			
Wheezing			

Lightheadedness on standing			
Fainting spells			
Changes in sweating (more or less)			
Nausea, vomiting, diarrhea, bloating or constipation			
Changes in color of hands or feet			
Urinary difficulties			

What is your current functional status in this post-acute COVID-19 phase?

Current Functional Status	Yes	No	Stage
No symptoms			I
No limitation but I feel some symptoms			II
I avoid some of my daily activities			III
I struggle to take care of myself			IV
I am in bed all or nearly all the time			V
I was hospitalized for COVID-related symptoms			Severe

*

Have you been vaccinated for COVID-19?

Vaccine Type	Select one below	Date, first dose	Date, second dose
BioNTech, Pfizer			
Moderna, NIAID			
Johnson & Johnson (JJ)			
No vaccination			

*modified scale from reference: Klok FA, Boon GJAM, Barco S, et al. The Post-COVID-19 Functional Status scale: a tool to measure functional status over time after COVID-19. Eur Respir J 2020; 56: 2001494

Supplementary Tables (2 and 3) **Supplementary Tables.**

Table 3. Distribution of Comorbid Condition on PASC Population.

Comorbidities	Number/Percentage
BMI $\geq$ 30	47 (35.1%)
Hypertension	24 (17.9%)
Chronic Lung Disease	17 (12.7%)
Diabetes Mellitus	9 (6.7%)
Cardiovascular Disease	4 (3%)
Immunosuppressive therapy	5 (3.7%)
HIV (+)	1 (0.7%)
Cirrhosis	1 (0.7%)

PASC: Post-Acute Sequela of SARS-Cov-2 infection

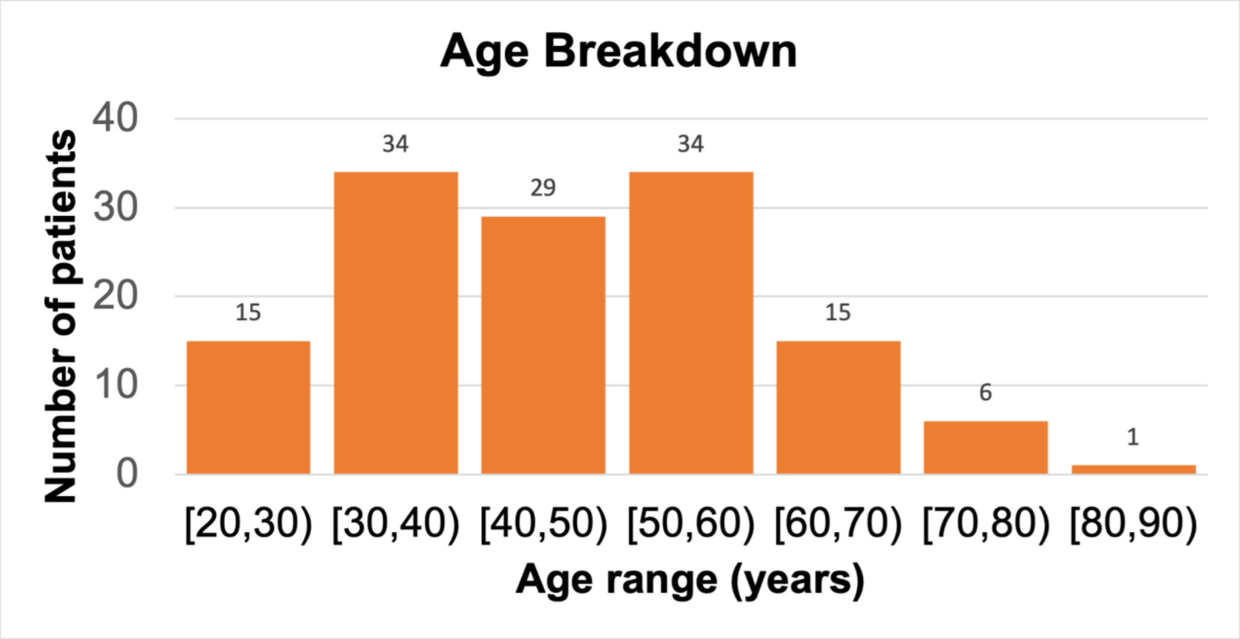
Age Breakdown
Number of patients
40
30
20
10
0
34
29
34
15
15
6
1
[20,30)
[30,40)
[40,50)
[50,60)
[60,70)
[70,80)
[80,90)
Age range (years)

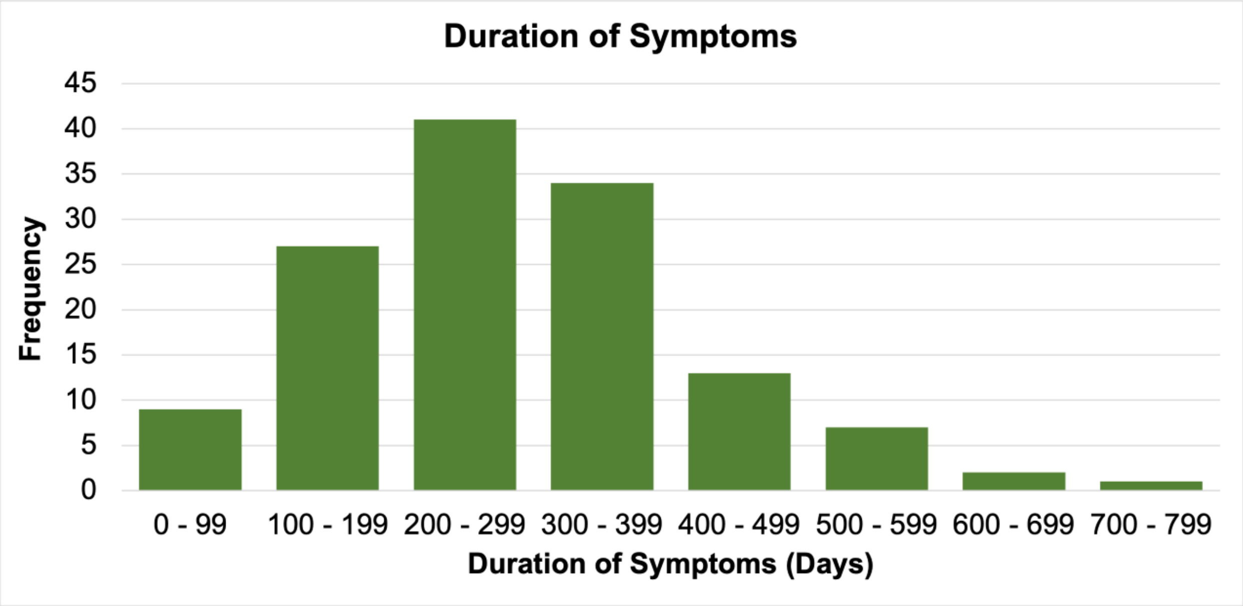
Duration of Symptoms
Frequency
45
40
35
30
25
20
15
10
5
0
0 - 99
100 - 199
200 - 299
300 - 399
400 - 499
500 - 599
600 - 699
700 - 799
Duration of Symptoms (Days)

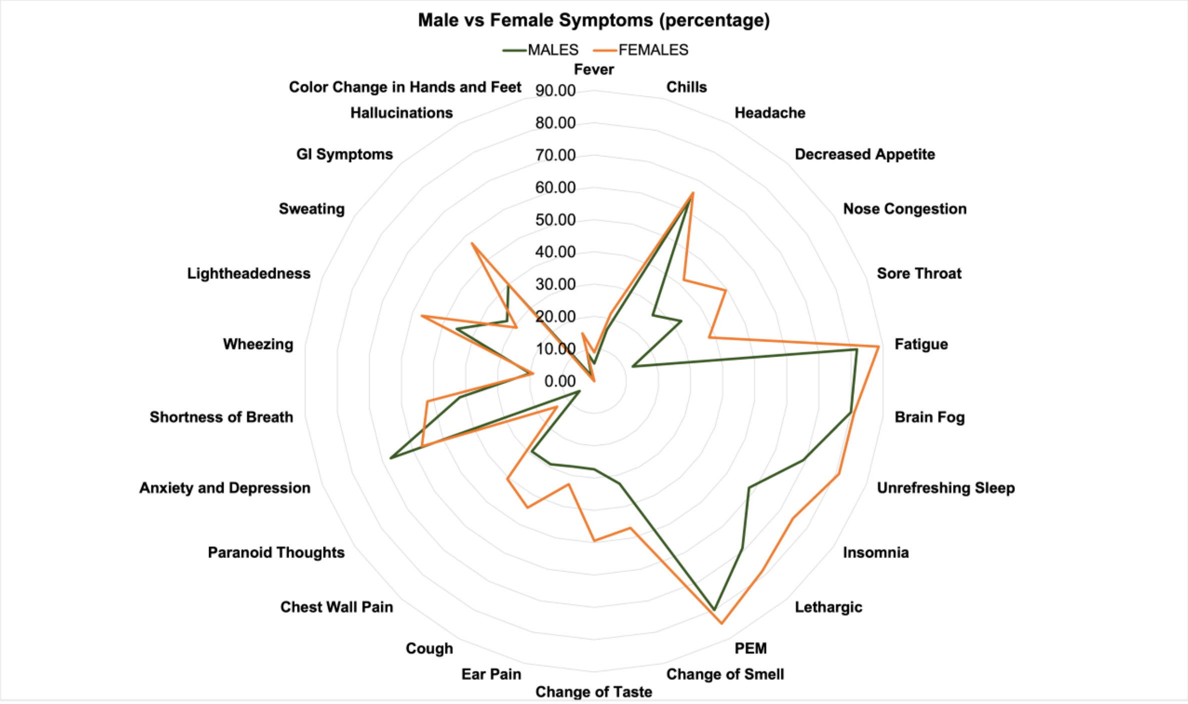
Male vs Female Symptoms (percentage)
MALES
FEMALES
Fever
Chills
Headache
Decreased Appetite
Nose Congestion
Sore Throat
Fatigue
Brain Fog
Unrefreshing Sleep
Insomnia
Lethargic
PEM
Change of Smell
Change of Taste
Ear Pain
Cough
Chest Wall Pain
Paranoid Thoughts
Anxiety and Depression
Shortness of Breath
Wheezing
Lightheadedness
Sweating
GI Symptoms
Hallucinations
Color Change in Hands and Feet
90.00
80.00
70.00
60.00
50.00
40.00
30.00
20.00
10.00
0.00

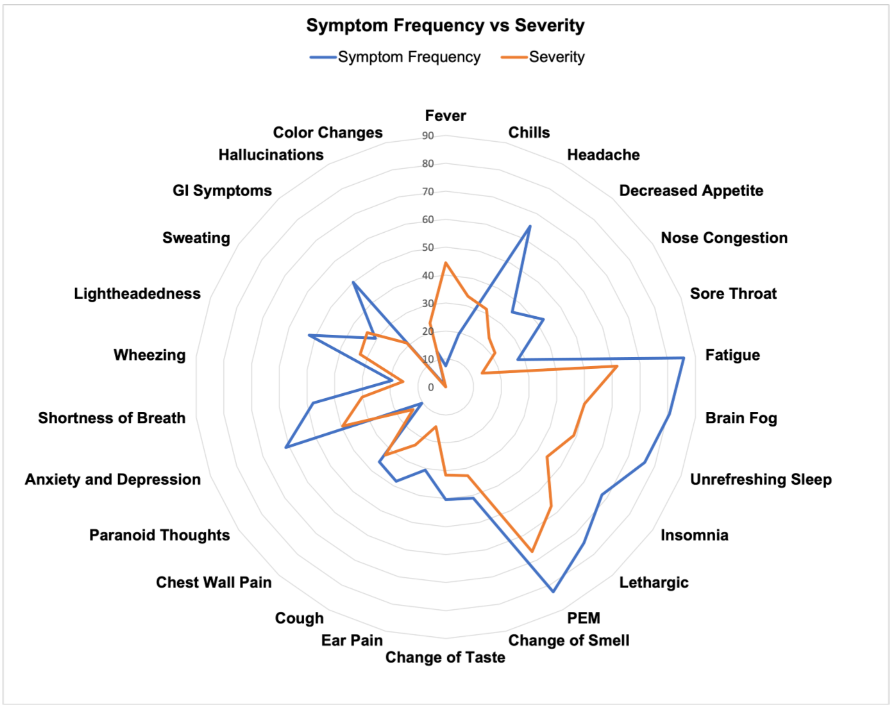
Symptom Frequency vs Severity
Symptom Frequency
Severity
Fever
Chills
Headache
Decreased Appetite
Nose Congestion
Sore Throat
Fatigue
Brain Fog
Unrefreshing Sleep
Insomnia
Lethargic
PEM
Change of Smell
Change of Taste
Ear Pain
Cough
Chest Wall Pain
Paranoid Thoughts
Anxiety and Depression
Shortness of Breath
Wheezing
Lightheadedness
Sweating
GI Symptoms
Hallucinations
Color Changes
90
80
70
60
50
40
30
20
10
0

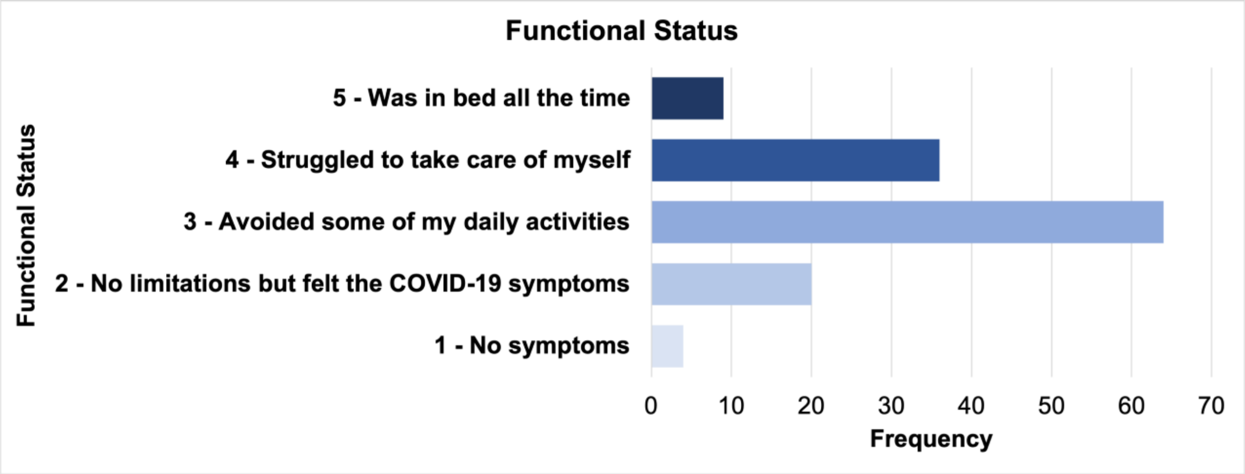
Functional Status
5 - Was in bed all the time
4 - Struggled to take care of myself
3 - Avoided some of my daily activities
2 - No limitations but felt the COVID-19 symptoms
1 - No symptoms
Functional Status
0
10
20
30
40
50
60
70
Frequency

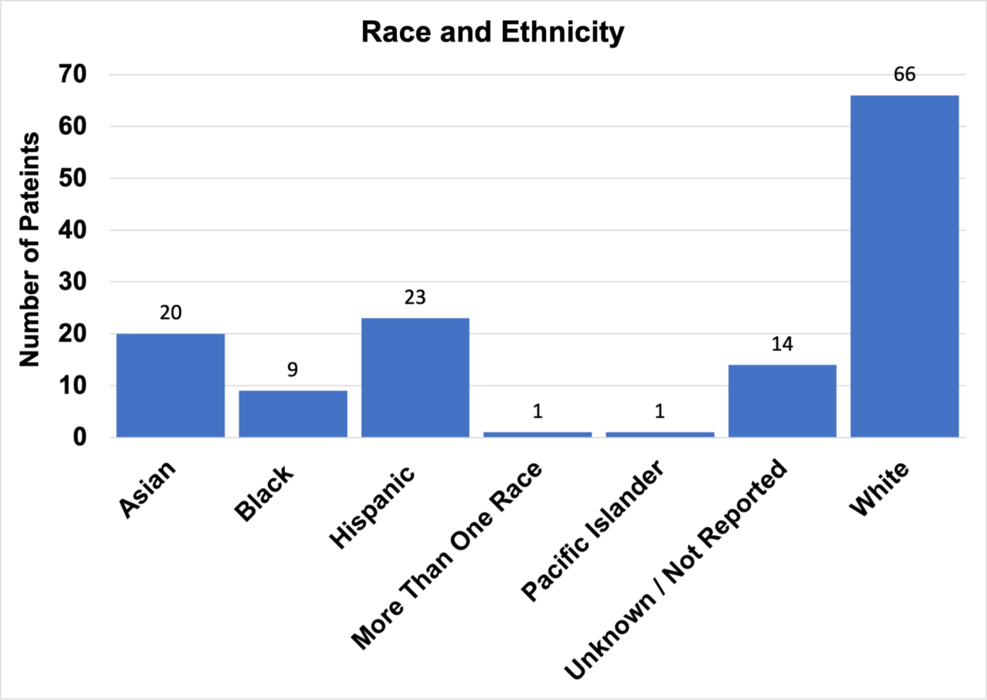
Race and Ethnicity
Number of Pateints
70
60
50
40
30
20
10
0
20
9
23
1
1
14
66
Asian
Black
Hispanic
More Than One Race
Pacific Islander
Unknown / Not Reported
White

13 most common symptoms

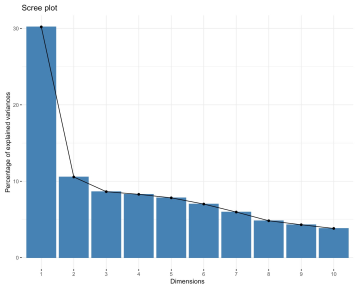

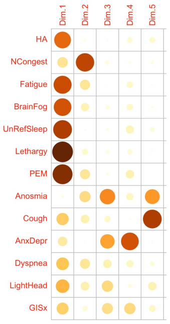

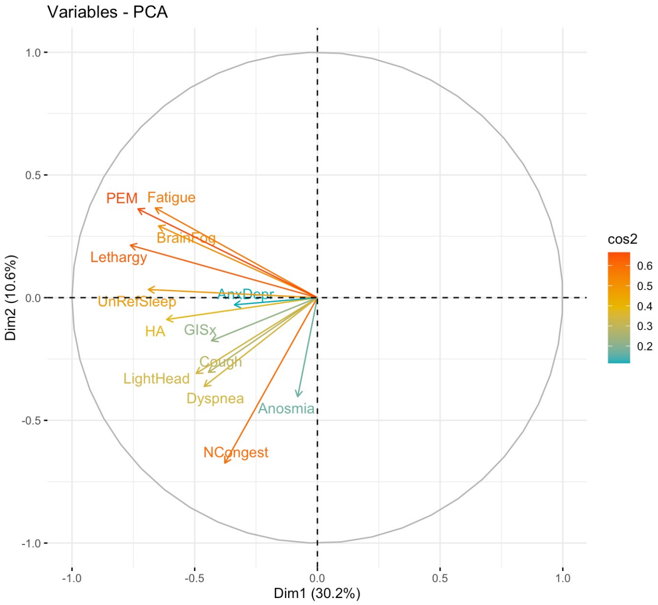

Total Symptoms, and distribution by sex

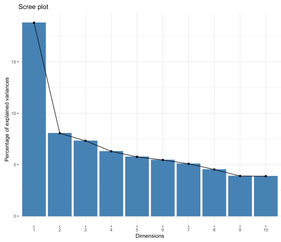

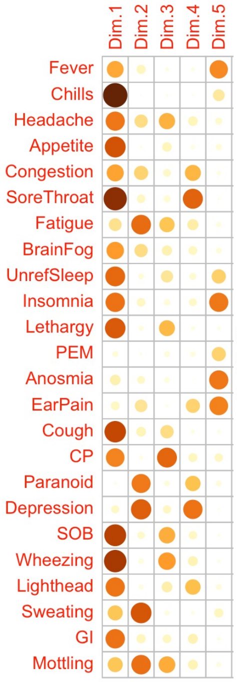

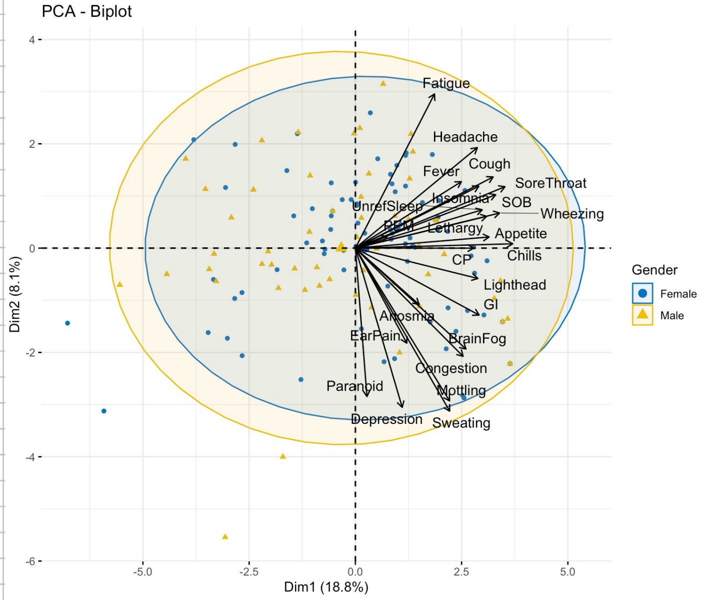

Common symptoms male:

	eigenvalue	variance.percent	cumulative.variance.percent
Dim.1	3.1318023	24.090787	24.09079
Dim.2	1.7423991	13.403070	37.49386
Dim.3	1.4808081	11.390832	48.88469
Dim.4	1.2178200	9.367846	58.25253
Dim.5	1.0799056	8.306966	66.55950
Dim.6	1.0063531	7.741177	74.30068
Dim.7	0.7773718	5.979783	80.28046
Dim.8	0.6786311	5.220239	85.50070
Dim.9	0.5662909	4.356084	89.85678
Dim.10	0.4300200	3.307846	93.16463
Dim.11	0.3619187	2.783990	95.94862
Dim.12	0.2912270	2.240208	98.18883
Dim.13	0.2354524	1.811172	100.00000

Common symptoms female:

	eigenvalue	variance.percent	cumulative.variance.percent
Dim.1	4.6530112	35.792393	35.79239
Dim.2	1.3126076	10.096982	45.88938
Dim.3	1.1123348	8.556422	54.44580
Dim.4	1.0321811	7.939854	62.38565
Dim.5	0.9694872	7.457594	69.84325
Dim.6	0.8592313	6.609471	76.45272
Dim.7	0.7104871	5.465285	81.91800
Dim.8	0.6237441	4.798032	86.71603
Dim.9	0.4749512	3.653471	90.36950
Dim.10	0.4092251	3.147885	93.51739
Dim.11	0.3378931	2.599177	96.11657
Dim.12	0.2790088	2.146221	98.26279
Dim.13	0.2258376	1.737212	100.00000

Common symptoms total:

	eigenvalue	variance.percent	cumulative.variance.percent
Dim.1	3.9263275	30.202520	30.20252
Dim.2	1.3727348	10.559499	40.76202
Dim.3	1.1229366	8.637974	49.39999
Dim.4	1.0777255	8.290196	57.69019
Dim.5	1.0180607	7.831236	65.52142
Dim.6	0.9121950	7.016885	72.53831
Dim.7	0.7759026	5.968481	78.50679
Dim.8	0.6279203	4.830156	83.33695
Dim.9	0.5602774	4.309826	87.64677
Dim.10	0.4973165	3.825512	91.47228
Dim.11	0.4072518	3.132706	94.60499
Dim.12	0.3895180	2.996292	97.60128
Dim.13	0.3118331	2.398717	100.00000

Eigenvalues 12 domains (Top total, Middle female and low male)

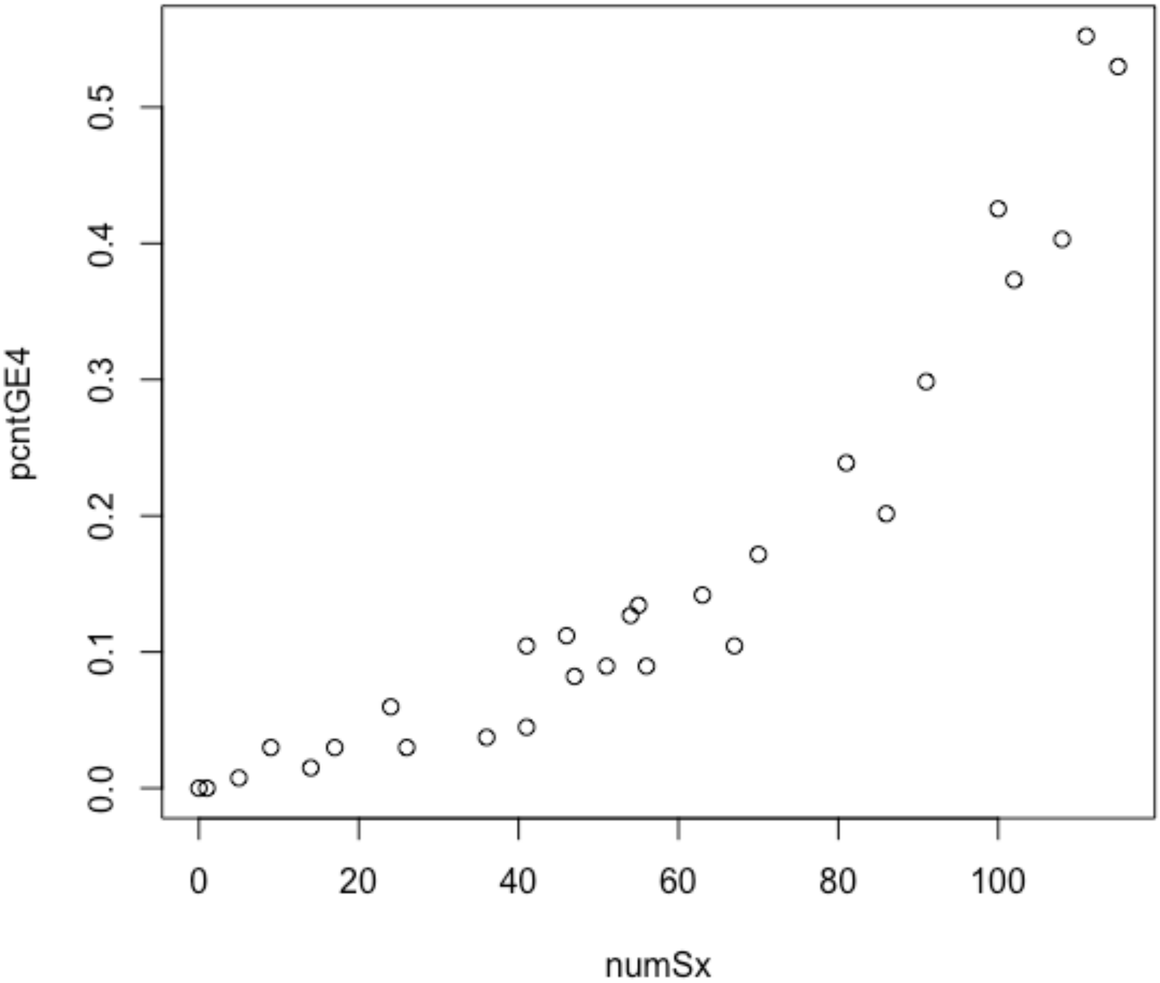

Parson Correlation Coefficient (PCC) frequency of 29 symptoms and severity (Likert scale 4 and 5).
