# Supplementary figures and images for: Myalgic Encephalomyelitis/Chronic Fatigue Syndrome is common in post-acute sequelae of SARS-CoV-2 infection (PASC): Results from a post-COVID-19 multidisciplinary clinic
Source: Front Neurol. 2023 Feb 24;14:1090747. doi: 10.3389/fneur.2023.1090747 (PMC9998690; doi:10.3389/fneur.2023.1090747)

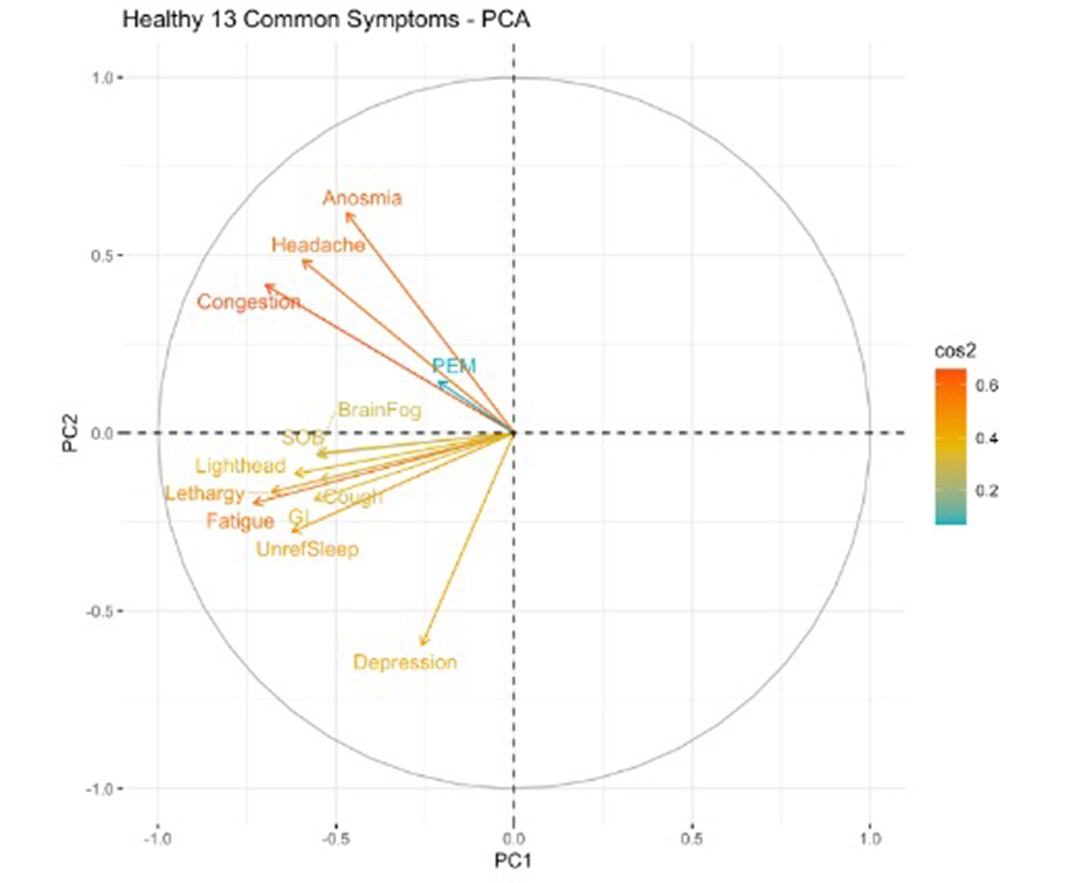

Supplement: Supplementary file 2 [file Image_1.JPEG]
